# A 40-Year-Old Female With Mycobacterium abscessus Successfully Treated With a Dual Beta-Lactam Combination

**DOI:** 10.7759/cureus.40993

**Published:** 2023-06-26

**Authors:** Natalia Moguillansky, Kathryn DeSear, Khalid M Dousa

**Affiliations:** 1 Department of Medicine, Division of Pulmonary Critical Care and Sleep Medicine, University of Florida Health, Gainesville, USA; 2 Department of Pharmacy, University of Florida Shands Hospital, Gainesville, USA; 3 Department of Infectious Disease, Case Western Reserve University School of Medicine, Cleveland, USA

**Keywords:** dual beta-lactams, nontuberculous mycobacteria, atypical pneumonia, pneumonia, imipenem, mycobacterium abscessus

## Abstract

Nontuberculous mycobacteria (NTM) infections are difficult to treat conditions, specially *Mycobacterium abscessus *(*Mab*) lung disease. The most recent ATS/ERS/ESCMID/IDSA clinical practice guidelines (2020) recommend regimens of multiple intravenous (IV) and oral antibiotics. Recent in vitro studies on *M. abscessus *show that the combination of two beta-lactam antibiotics, as well as select beta-lactamase inhibitors, provides significant synergy in its treatment. We present the first in vivo case of the successful treatment of *Mycobacterium abscessus* with imipenem and amoxicillin in addition to macrolides, clofazimine, and inhaled liposomal amikacin.

## Introduction

Nontuberculous mycobacteria (NTM) pulmonary disease is a difficult condition to diagnose and treat. The most recent ATS/ERS/ESCMID/IDSA clinical practice guidelines (2020) for the treatment of NTM include clinical, radiographic, and microbiologic criteria. These consist of pulmonary or systemic symptoms and nodular or cavitary opacities on chest imaging, as well as two positive culture results on two separate expectorated sputum samples or at least one bronchial wash or lavage or lung biopsy showing granulomatous inflammation with positive acid-fast bacilli (AFB).

Recent in vitro studies on *Mycobacterium abscessus* (*Mab*) showed that the combination of two beta-lactam antibiotics, as well as select beta-lactamase inhibitors, provides significant synergy. We present the first in vivo case of the successful treatment of *Mab* with imipenem and amoxicillin in addition to macrolides, clofazimine, and inhaled liposomal amikacin.

## Case presentation

A 40-year-old female of Caucasian ethnicity with a medical history of common variable immunodeficiency (CVID) and obstructive sleep apnea (OSA) treated with continuous positive airway pressure (CPAP) presented to the pulmonary clinic with a complaint of cough. She was diagnosed with CVID at the age of 30 and had been receiving intravenous immunoglobulin (IVIG) therapy for several years. She had no history of cigarette smoking but had a significant family history of asthma, diabetes, and psoriasis.

**Figure 1:**
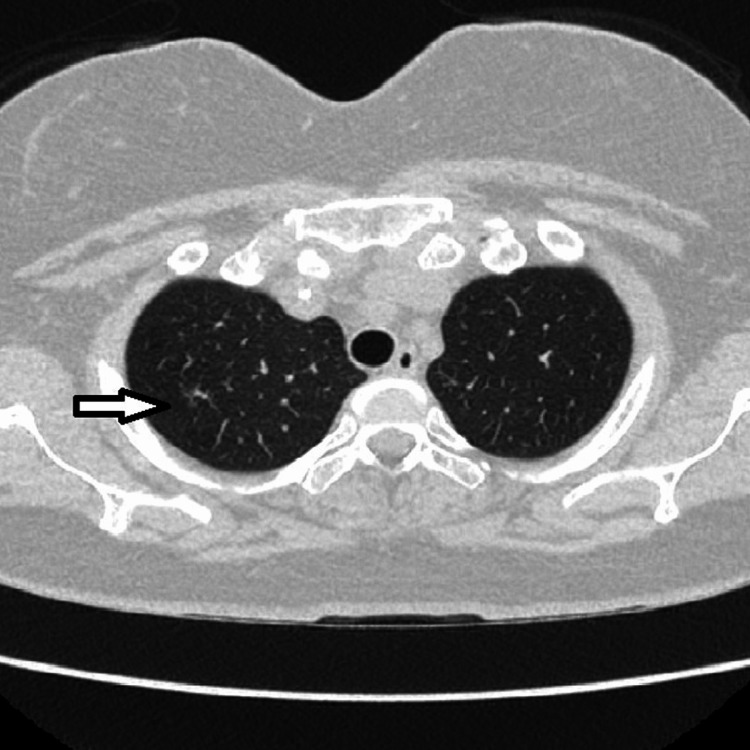
CT of the chest. Lung window. Lung upper lobes. Right upper lobe tree in bud opacities (arrow).

**Figure 2:**
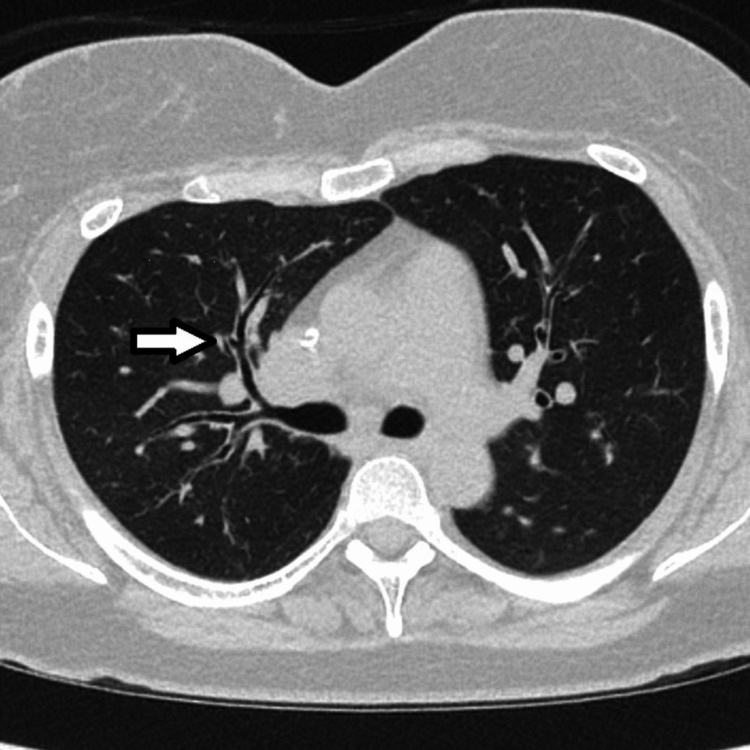
CT of the chest. Lung window. A level below the carina. Right middle lobe bronchiectasis (arrow).

**Figure 3:**
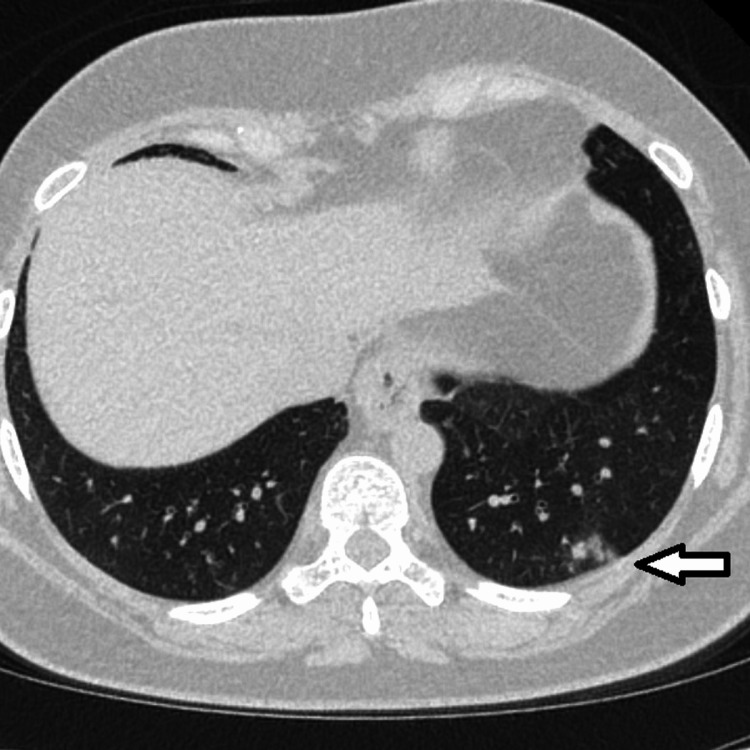
CT of the chest. Lung window. Lung lower lobes. Left lower lobe nodules (arrow).

She had a one-year history of dyspnea with moderate exertion, wheezing, and productive cough. She had six respiratory exacerbations treated with steroids (brief several-day courses) and antibiotics in the year prior to presentation, without the improvement of her respiratory symptoms. On presentation, her oxygen saturation was 95% on room air, and her blood pressure was 121/83 mmHg with a pulse of 87 beats per minute and a body mass index of 35 kg/m^2^. On general examination, she was in no acute respiratory distress and was fully alert and oriented. Her lungs were clear to auscultation bilaterally, and the general examination of both the cardiac and nervous systems was unremarkable. A CT scan of the chest showed scattered foci of bronchocentric nodules primarily within the right upper lobe, right middle lobe bronchiectasis, and left lower lobe nodules. There were no dominant pulmonary nodules or evidence of cavitation. There was scattered mosaic attenuation throughout both lungs, consistent with air trapping (Figures 1-3 and Video 1). She was initiated on airway clearance with albuterol and 3% nebulized saline, as well as a flutter valve twice a day.

**Video 1:** CT of the chest. Lung window. Right upper lobe tree in bud opacities. Right middle lobe bronchiectasis. Left lower lobe nodules.

Subsequently, she underwent bronchoscopy with bronchoalveolar lavage culture that identified the growth of *Mab* subspecies *massiliense*. The *Mab* was susceptible to amikacin (minimum inhibitory concentration {MIC} of <16 mcg/ml) and clarithromycin (MIC of 0.25 mcg/ml). A cell-based assay was conducted at Case Western Reserve University School of Medicine to assess the antimicrobial susceptibility of the clinical isolate to a dual β-lactam combination. Of note, a MIC for imipenem of less than 4 mcg/ml is susceptible, between 4 and 16 mcg/ml is intermediate, and more than 16 mcg/ml is resistant. The initial test results revealed a MIC of 32 mcg/ml for imipenem alone. However, the addition of amoxicillin significantly improved the imipenem MIC to 4 mcg/ml. Moreover, ceftaroline further increased the susceptibility of imipenem to a MIC of 0.2 mcg/ml. Similarly, the addition of cefuroxime resulted in a reduced imipenem MIC of 0.25 mcg/ml. Additionally, the serial dilution of imipenem with cefdinir demonstrated improved susceptibility, with a MIC of less than 0.12 mcg/ml. When relebactam was added to the imipenem + amoxicillin combination, the resultant imipenem MIC was <0.12 mcg/ml. Bacterial and fungal cultures were negative, as well as *Aspergillus* galactomannan antigen.

Based on the above findings and to enhance *Mab* susceptibility to imipenem, therapy with imipenem-cilastatin intravenous (IV) 1000 mg twice/day and amoxicillin 500 mg twice/day was initiated and timed to be given alongside with clofazimine 100 mg daily, azithromycin 250 mg daily, and liposomal amikacin inhalation suspension. Even though clofazimine is usually used in refractory cases, it was chosen over other oral drugs due to the susceptibility’s patterns. Imipenem and amoxicillin were discontinued after two months, and she continued with the remaining all-oral and inhaled regimen.

**Figure 4:**
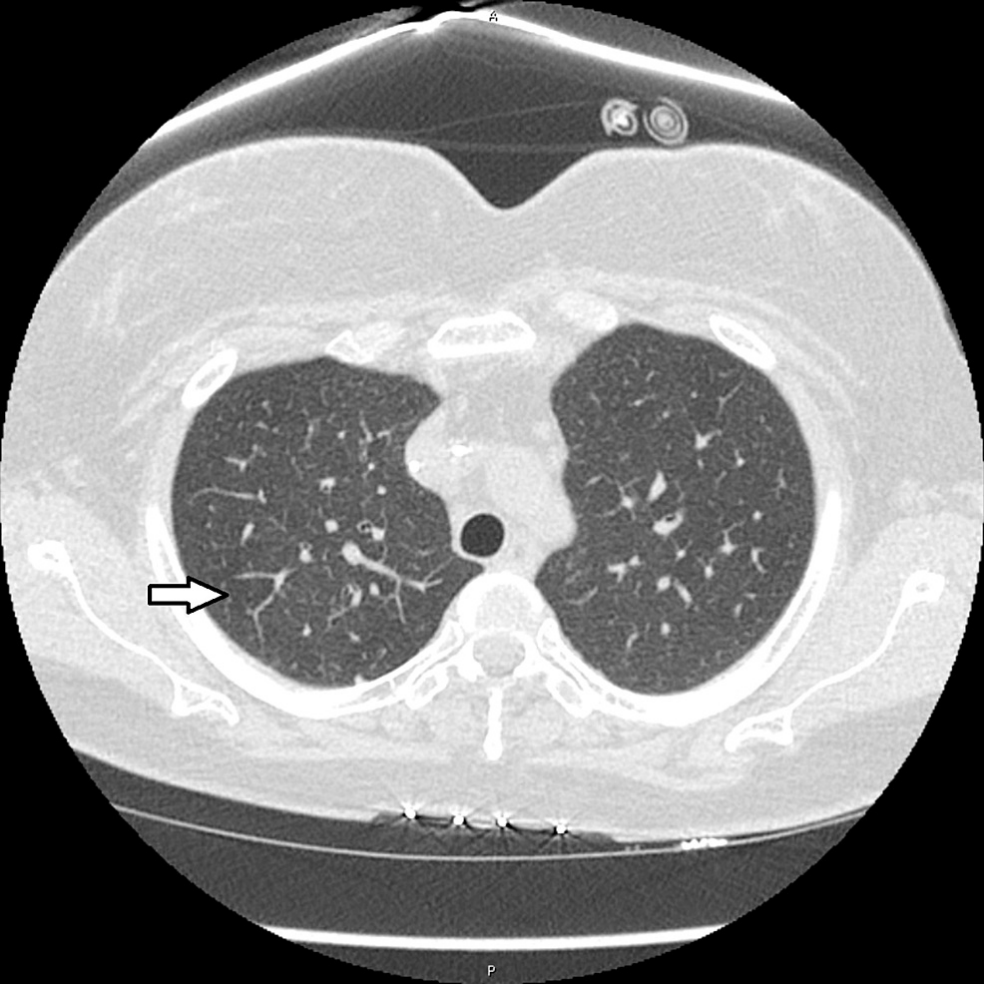
CT of the chest. Lung window. Lung upper lobes. The improvement of the right upper lobe tree in bud opacities (arrow). Compare with Figure 1.

**Figure 5:**
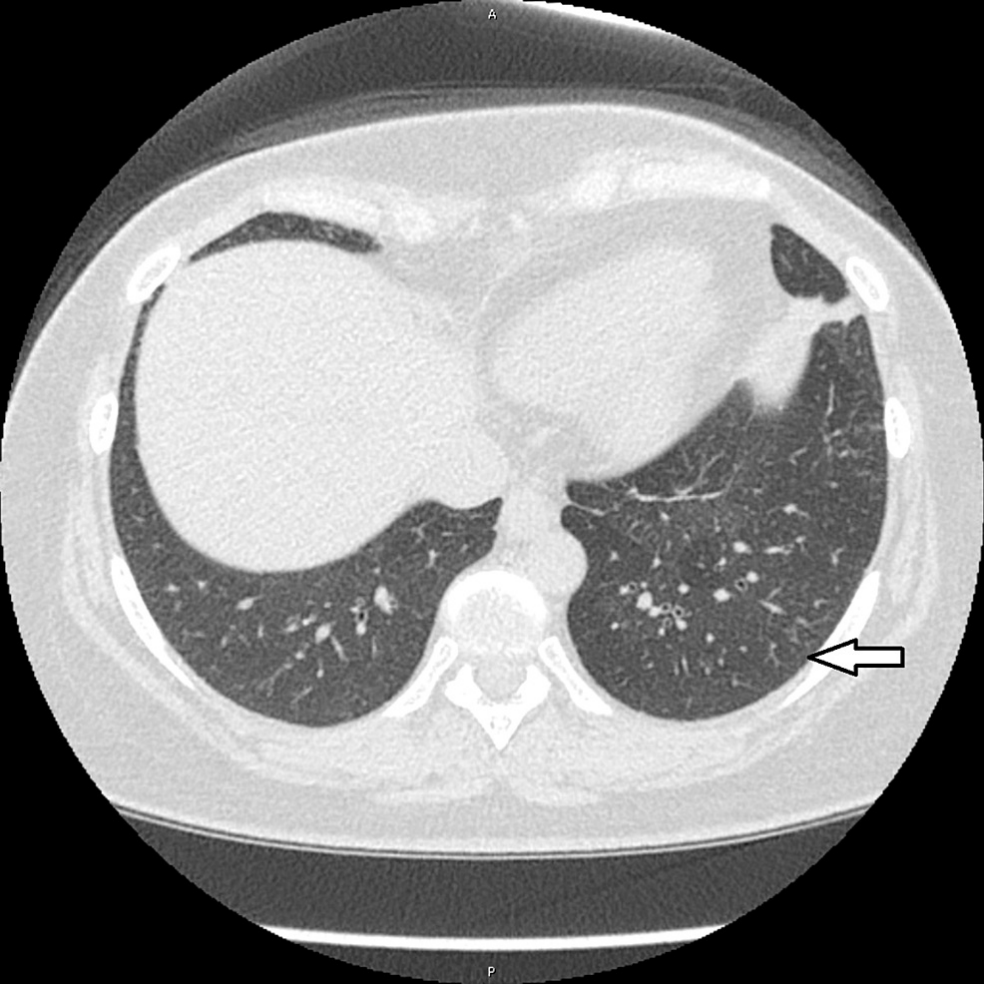
CT of the chest. Lung window. Lung lower lobes. The improvement of the left lower lobe nodules (arrow). Compare with Figure 3.

Because she was unable to produce sputum, a bronchoscopy with bronchoalveolar lavage was repeated five months after starting therapy. It revealed negative AFB, bacterial, and fungal cultures. Her repeat CT scan of the chest after five months of therapy showed interval decrease in the size and number of multiple bronchocentric nodules primarily within the right upper lobe and the near-complete resolution of previously noted bronchocentric nodules in the posterior segment left lower lobe (Figures 4-5). The overall CT scan was improved from the previous one (Video 2).

**Video 2:** Follow-up CT of the chest after treatment. The resolution of the right upper lobe tree in bud opacities and the left lower lobe nodules.

She also had improvement of her symptoms of cough, dyspnea, and wheezing. She will complete 12 months of treatment with azithromycin, clofazimine, and amikacin liposome inhalation suspension after her first negative AFB culture.

## Discussion

NTM pulmonary disease is a difficult condition to diagnose and treat. Epidemiologic studies in North America show that *Mab* complex is the second most common NTM isolated after *Mycobacterium avium* complex [1]. The classic presentation of NTM includes cough, sputum, and hemoptysis, as well as systemic symptoms such as fatigue, night sweats, and weight loss. The common radiographic findings of NTM are reticulonodular opacities bilaterally, branching nodular opacities (tree in bud pattern), and bronchiectasis. Air space consolidations and cavities are seen less frequently [2].

The most recent ATS/ERS/ESCMID/IDSA clinical practice guidelines (2020) divide the treatment for *Mab* pulmonary disease between macrolide-susceptible and macrolide-resistant organisms [3]. For macrolide-susceptible organisms, the guidelines recommend an initial phase of 1-2 parenteral drugs (amikacin, imipenem, cefoxitin, and tigecycline) and two oral drugs (azithromycin, clofazimine, and linezolid), as well as inhaled amikacin [3]. For macrolide-resistant organisms, they recommend an initial phase of 2-3 parenteral drugs (amikacin, imipenem, cefoxitin, and tigecycline) and 2-3 oral drugs (azithromycin, clofazimine, and linezolid) and inhaled amikacin. Azithromycin is added for its immunomodulatory effect [3]. Treatment should be continued for one year after the first negative culture. Even though the use of dual β-lactams is not mentioned in the current guidelines, we followed these guidelines for all the other drugs used. Of note, nonadherence to current guidelines may be detrimental for patients with NTM [4].

Novel in vitro studies have shown that the use of dual β-lactam agents, with and without β-lactamase inhibitors, has significant potential in the treatment of *Mab* complex. Beta-lactams exert their activity by inhibiting the synthesis of an essential component of the bacterial cell wall, the peptidoglycan [5]. It has been hypothesized that two β-lactams that inhibit distinct sets of nonredundant enzymes may exhibit synergy in antibacterial activity, based on the differential inhibition of enzymes involved in peptidoglycan synthesis [6].

A recent in vitro study on *Mab* by Lopeman et al. demonstrated that the addition of amoxicillin reduces the minimum inhibitory concentration of imipenem-relebactam by fourfold [7]. Another in vitro study on *Mab* by Pandey et al. evaluated the in vitro activities of ceftaroline and imipenem against 30 strains of *Mab* in the presence of the combination drug ceftazidime plus avibactam. The addition of these drugs further decreased the MIC of imipenem and ceftaroline by fourfold [8]. Story-Roller et al. studied the effect of β-lactam combinations in 21 strains of *Mab*; they found that the combination of cefoxitin and imipenem exhibited synergy against all 21 of the clinical strains tested, maintaining efficacy despite vast differences in drug resistance profiles [6]. Also, an in vitro study by Dousa et al. on *Mab* showed that the addition of durlobactam, a recently developed class of β-lactamase inhibitor, provides the protection of amoxicillin and imipenem against hydrolysis in *Mycobacterium* species [9].

The successful treatment of our patient with a dual β-lactam combination as part of a multi-drug regimen highlights the remarkable potential of this approach in targeting *Mab* infections. The addition of amoxicillin to imipenem amplified the activity of the latter, which targets bacterial transpeptidation and halts cell wall synthesis. Although clinical data on the effectiveness of this combination therapy are limited, as stated above, recent in vitro studies have reported significant synergy between two β-lactams, as well as between β-lactams and β-lactamase inhibitors against *Mab*.

The main limitation this case faces is that the *Mab* has a subspecies *massiliense*, which has a higher proportion of patients with sputum conversion and negative cultures (88%) compared to subspecies *abscessus* (25%). It is possible that the positive outcome observed in this patient was due to the combination of factors such as the subspecies being *massiliense*, the multi-drug regimen, and the fact that the isolate was susceptible to macrolides, which typically have a high success rate [3].

To our knowledge, our case report is the first to show the successful treatment outcome of dual β-lactam therapy, underscoring the tremendous promise of this approach for patients suffering from *Mab* infections. Further research is warranted to assess the effectiveness of this approach and explore its potential for improving outcomes in patients with NTM pulmonary disease.

## Conclusions

NTM and especially *Mab* pulmonary diseases are difficult to diagnose and treat conditions. Once the diagnosis is made, the guidelines recommend multiple oral and IV therapies depending on macrolide resistance, including macrolides, aminoglycosides, β-lactam, and other class drugs. The use of dual β-lactam in the treatment of *Mab* infections is still an emerging area of research. There are recent in vitro studies on *Mab* that show that the combination of two β-lactams provides significant synergy. However, to date, there are no published in vivo studies that demonstrated the success of the use of the combination of two β-lactams. We present the first in vivo case of treatment success by combining two β-lactams plus other standard NTM drugs to treat *Mab* lung disease.

## References

[1] Prevots DR, Marras TK (2015). Epidemiology of human pulmonary infection with nontuberculous mycobacteria: a review. Clin Chest Med.

[2] Han D, Lee KS, Koh WJ, Yi CA, Kim TS, Kwon OJ (2003). Radiographic and CT findings of nontuberculous mycobacterial pulmonary infection caused by Mycobacterium abscessus. AJR Am J Roentgenol.

[3] Daley CL, Iaccarino JM, Lange C (2020). Treatment of nontuberculous mycobacterial pulmonary disease: an official ATS/ERS/ESCMID/IDSA clinical practice guideline. Eur Respir J.

[4] Adjemian J, Prevots DR, Gallagher J, Heap K, Gupta R, Griffith D (2014). Lack of adherence to evidence-based treatment guidelines for nontuberculous mycobacterial lung disease. Ann Am Thorac Soc.

[5] Story-Roller E, Maggioncalda EC, Cohen KA, Lamichhane G (2018). Mycobacterium abscessus and β-lactams: emerging insights and potential opportunities. Front Microbiol.

[6] Story-Roller E, Galanis C, Lamichhane G (2021). Β-lactam combinations that exhibit synergy against Mycobacteroides abscessus clinical isolates. Antimicrob Agents Chemother.

[7] Lopeman RC, Harrison J, Rathbone DL, Desai M, Lambert PA, Cox JA (2020). Effect of amoxicillin in combination with imipenem-relebactam against Mycobacterium abscessus. Sci Rep.

[8] Pandey R, Chen L, Manca C (2019). Dual β-lactam combinations highly active against Mycobacterium abscessus complex in vitro. mBio.

[9] Dousa KM, Nguyen DC, Kurz SG (2022). Inhibiting Mycobacterium abscessus cell wall synthesis: using a novel diazabicyclooctane β-lactamase inhibitor to augment β-lactam action. mBio.

